# Uncovering leaf and root microbiomes of mangrove trees in French Guiana

**DOI:** 10.3389/frmbi.2026.1782119

**Published:** 2026-04-22

**Authors:** Mathilde Vigneron, Sébastien Halary, Sandrine Crochemore, Laetitia Plaisance, Nathalie Parthuisot, Yvan Bettarel

**Affiliations:** 1 MARBEC, Université de Montpellier, CNRS, Ifremer, IRD, Place Eugène Bataillon – Bat 24, UMR 9190, Montpellier, France; 2 Molécules de Communication et Adaptation des Microorganismes, Muséum National d’Histoire Naturelle, UMR 7245 CNRS, Paris, France; 3 Centre de Recherche sur la Biodiversité et l’Environnement (CRBE), Université de Toulouse, CNRS, IRD, Toulouse INP, Université Toulouse 3 – Paul Sabatier (UT3), UMR 5300, Toulouse, France

**Keywords:** bacteria, French Guiana, leaf, mangrove, microbiome, root

## Abstract

Microorganisms are now widely acknowledged as essential contributors to the health and resilience of coastal environments. Yet, mangrove ecosystems, despite offering numerous ecological and economic services, remain relatively overlooked in microbial research. In this study, we examined the bacteriome of the rhizosphere and the phyllosphere of two mangrove tree species: *Avicennia germinans* and *Rhizophora mangle*. Both species were sampled along the banks of the Sinnamary estuary in French Guiana. Our results revealed notable differences in microbiome composition between the two organs and between the two tree species. On average, only 0.3% of ASVs were shared between the leaves and roots and 2.2% between *A. germinans* and *R. mangle*. The taxonomic differences were characterized mainly by the significant presence of Rhodothermia and Bacteroidia in the leaves and Cyanobacteria and Planctomycetia in the roots. Furthermore, our results showed that the root microbiome of both species was only weakly influenced by the surrounding water and sediment, with an average of less than 0.7% of ASVs shared. Finally, our study indicates a strong specificity in the bacterial communities of both the phyllosphere and rhizosphere and also raises questions regarding the near absence of Gammaproteobacteria in both the leaves and roots, which remain to be elucidated.

## Introduction

Thanks to their biological richness and diversity, mangrove forests provide a large number of ecosystem services and play key ecological roles on a global scale (Dabalà et al., 2023). Recently, these tropical intertidal ecosystems have attracted the attention of microbiologists, who are focusing their efforts on understanding their role in maintaining the health and productivity of mangroves. Through their involvement in processes such as nutrient cycles and organic matter or pollutant production and degradation, microorganisms have been revealed to play a leading role in mangrove ecosystems (Ding et al., 2025; Holguin et al., 2001; Meng et al., 2022). They are also involved in many interactions with mangrove trees and support essential functions for host survival as epiphytic or endophytic colonizers (Elyamine et al., 2023). However, the majority of microbiological studies conducted thus far have focused on two specific environmental matrices in mangroves: soil and seawater. Meanwhile, the microbial assemblages associated with local plant and animal hosts remain poorly characterized, despite their potentially critical roles in such ecosystems.

In this study, we explored the microbiome of mangrove trees, which are keystone species given their role in carbon sequestration, providing a habitat for local fauna, stabilizing sediments, protecting coastlines, etc. (D. Alongi, 2002; Kathiresan and Bingham, 2001). More specifically, our aim was to examine variability in microbiome composition: i) between two emblematic mangrove tree species: *Avicennia germinans* and *Rhizophora mangle*, two of the most widespread mangrove tree species worldwide (Kumar et al., 2021); and ii) between two major organs: leaves and roots—the leaves, due to their role in decomposition and nutrient cycling, their influence on the tree’s resistance to pathogens and environmental stress, and their linking role between aboveground plant processes and the surrounding environment facilitating the transfer of organic matter through litter fall; and the roots, which represent a critical transition zone between the tree, the surrounding water, and the sediment, thereby acting as a key interface for microbial colonization. Finally, iii) we also examined whether potential links exist between the mangrove tree microbiome and that of environmental matrices (water and soil). The study site was located in the estuary of the Sinnamary River in French Guiana, where the mangrove is among the most dynamic in the world and remains largely unaffected by human activities (Marchand et al., 2006).

## Materials and methods

### Sampling procedure

The samples were collected along the banks of the Sinnamary River estuary, French Guiana (5°26′54″N, 53°00′01″W). The site is part of a pristine area of the Amazon Basin with few anthropogenic activities and presents very low salinity and highly turbid water, due to the river’s influence (Fromard et al., 2004). Leaf and root samples were collected on *Avicennia germinans* and *Rhizophora mangle* mangrove trees. In order to minimize the collection of water or sediment bacterial communities when swabbing tree roots, pneumatophores of *A. germinans* and stilt roots of *R. mangle* were both sampled on their aerial parts. The bacteriome of each organ was collected in triplicate by swabbing the leaves and roots of six different individuals per species, using buccal swabs (SK-2S, Isohelix, Harrietsham, UK). Three individual trees were swabbed independently for their leaves and three others for their roots; they were then treated as biological replicates in downstream analyses. Trees of both species sampled were approximately 300 m apart, and this distance is considered to represent spatial independence. Triplicate samples of surrounding water (1 L, subsurface) and sediment (8–10 g, taken from the top 3 cm) were also collected from the sampling area of each species of mangrove tree.

### DNA extraction, amplification, and sequencing

Bacterial DNA was extracted using the DNeasy PowerSoil Pro Kit (QIAGEN^®^, Hilden, Germany). For root and leaf samples, extraction was processed directly from the entire swabs (triplicates). The extraction of bacterial DNA from water and sediment samples was carried out in triplicate using 0.2-μm porosity polycarbonate filter membranes (∅47 mm, Whatman, Nucleopore, Maidstone, UK) for the former after filtering 500 mL of seawater and from 5 g of raw sediment for the latter. Negative controls were obtained by performing blank extraction (*n* = 2). DNA quality and quantity were assessed by spectrophotometry (NanoDrop^®^, Wilmington, DE, USA). The V3–V4 region of the 16S rDNA gene was amplified using universal bacterial primers modified for Illumina sequencing: 341F* - CCTACGGGNGGCW GCAG/805R* - GACTACHVGGGTATC TAATCC (Klindworth et al., 2013). Successfully amplified samples as well as negative controls were sequenced on an Illumina platform (Fasteris^®^, Genesupport SA - Life Sciences, Plan-les-Ouates, Switzerland) using a 2 × 250-bp MiSeq platform.

### Bacterial sequencing processing

A total of 4, 323, 853 FASTQ format reads were obtained. Raw reads were processed with the open-access QIIME2 2024.10 software (Bolyen et al., 2019). The demultiplexed paired-end forward and reverse sequences were truncated based on the quality profiles. Sequences were then filtered, trimmed, and merged into 15, 855 amplicon sequence variants (ASVs), using the DADA2 pipeline implemented in QIIME2. After filtering and trimming, the samples contained 31, 217 ± 7, 851 reads (mean ± SD) per sample. Chimaeras and the relative abundance of ASVs assigned to non-prokaryotes, archaea, chloroplasts, and mitochondria were removed, as well as the ASVs found in the negative sequencing controls. Sequences were then aligned with the 16S Greengenes 2022.10 database (McDonald et al., 2024).

### Data analysis

Bacterial taxonomic diversity was then assessed with RStudio (R version 4.4.1, RStudio version 2025.05.0), using the *phyloseq* package (McMurdie and Holmes, 2013). Sequenced biological triplicates of each compartment and species were pooled for analyses. To assess microbial diversity among samples, abundances were pooled at the class taxonomic level. For each sample, the 20 most abundant classes were selected, and rare taxa with abundance <1% in each sample were removed. This arbitrary threshold was chosen for clarity, to facilitate visualization of dominant communities by reducing background noise associated with rare communities. We recognize, however, that these rare communities, despite their very low abundance, are likely to play an important ecological role in marine ecosystems (Troussellier et al., 2017). Results were graphically represented using the *ggplot2* package (Wickham, 2016). Bray–Curtis distance between the samples was calculated based on the dissimilarity of their microbial composition (Lozupone et al., 2007). To test for significant differences in the microbial composition between the leaves and roots and between tree organs and the environment, permutational multivariate analyses of variance (PERMANOVAs; Anderson, 2017) with 999 permutations were performed in RStudio, using the *vegan* package (Oksanen et al., 2025).

## Results and discussion

### The microbiome of mangrove trees: leaves vs. roots

**Figure 1:**
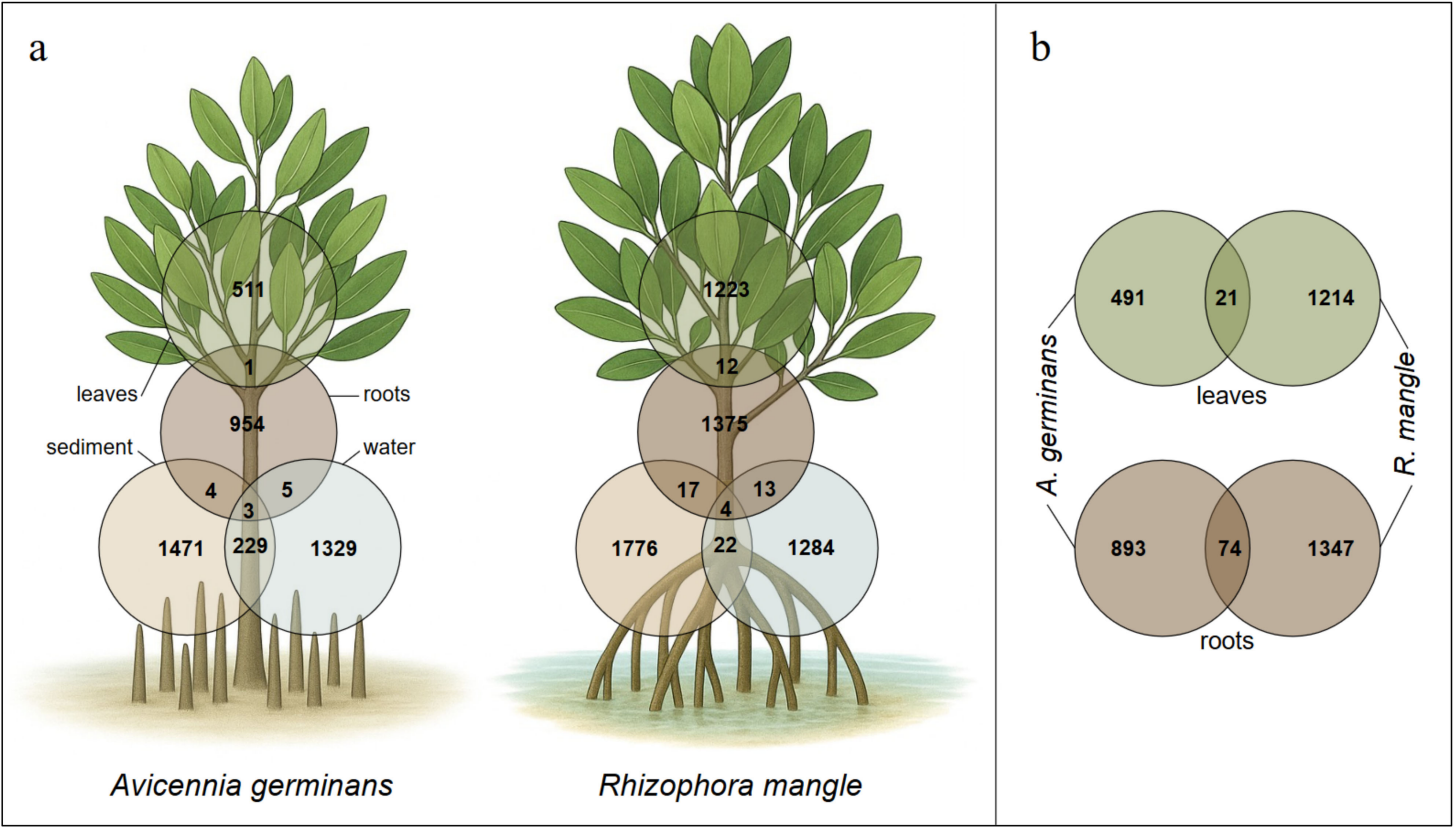
**(A)** Venn diagrams showing the number of shared and specific pooled ASVs among the different sampling compartments (leaves, roots, sediment, water) in *Avicennia germinans* and *Rhizophora mangle*. **(B)** Venn diagram of ASVs shared between the leaves and roots of both tree species.

**Figure 2:**
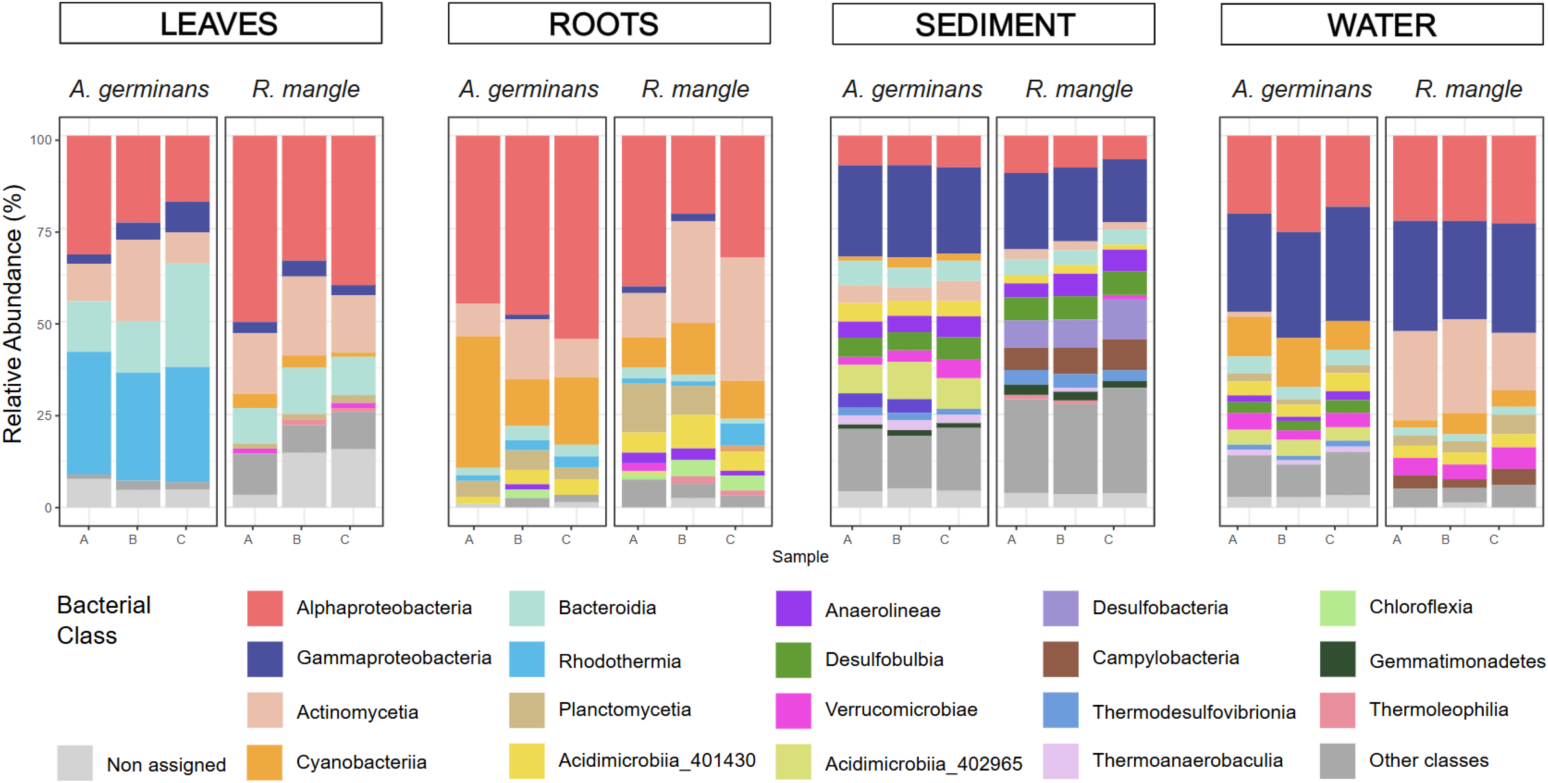
Relative abundance of bacterial classes in the different compartments, among the 20 more abundant taxa classes. Non-assigned ASVs after the assignment step are gathered in the “non-assigned” category. Taxa classes with a mean relative abundance <1% in each sampling site have been removed.

In general, roots harbored a greater number of ASVs than leaves, although this pattern was more pronounced in *A. germinans* (*m*_roots_ 967 > *m*_leaf_ 512) than in *R. mangle* (*m*_roots_ 1, 421 > *m*_leaf_ 1, 235) (**Figure 1**). The composition of bacterial communities also showed substantial differences between the two organs (**Figures 1**, **2**). Indeed, only a very small proportion of ASVs were shared between rhizosphere and phyllosphere in both *A. germinans* (*m* = 0.06%) and *R. mangle* (*m* = 0.45%). While the Alphaproteobacteria and Actinobacteria classes were broadly represented in both leaves and roots, our results also revealed a marked organ-specific bacterial signature. Such differences are not surprising given that the microbiomes of mangrove leaves and roots are subject to radically different ecological and functional conditions. The root microbiome mainly reflects the tree’s nutritional needs and tolerance to salt/anaerobic stress, while the leaf microbiome is rather structured by aerial factors (UV, drought, secondary metabolites) and by the need to protect the leaf surface (Hsiao et al., 2024; Zhuang et al., 2020).

*Leaves.* The most prominent trends in the leaves included a higher relative abundance of Bacteroidia, representing 13.0% of major taxa compared to 2.5% in the roots (**Figure 2**). Commonly found in mangrove environments (Law et al., 2019; Wang et al., 2024) and particularly on mangrove tree leaves (Elyamine et al., 2023), these bacteria are known to participate in the degradation of xenobiotic compounds (Ghose et al., 2024). This class is also known to inhabit human animal guts as a commensal but also as a potential pathogen for some species (Pan et al., 2023). Rhodothermia were also reported as a dominant group in the leaves, although this was more pronounced for *A. germinans* (**Figure 2**), and 60% of the ASVs assigned to Rhodothermia here belong to the Rubricoccaceae family, known to inhabit marine, saline, and thermophilic environments (Park et al., 2011, Park et al., 2013; Song et al., 2016). The *Rubrivirga* genus, which here represents 88.9% of the ASVs assigned to Rubricoccaceae, was previously found on mangrove tree leaves, where it could be involved in nutrient cycling, thanks to its degradation capacity of organic chemicals (Wainwright et al., 2023). Bacteria of the Rhodothermia class are known for their ability to degrade complex organic polymers such as cellulose and lignin (Dobruchowska et al., 2022; Okano et al., 2014), which are found in mangrove leaves. Their occurrence may therefore contribute significantly to nutrient cycling and carbon turnover in mangrove ecosystems.

*Roots.* The root microbiome was mostly characterized by a very high representation of Cyanobacteria and Planctomycetia (15.4% and 6.0% of the overall roots’ main taxa, respectively) compared to the leaves (1.8% and 1.2%). Both classes are known for their ability to colonize either freshwater or shallow-water marine environments and intertidal environments such as mangroves (Alvarenga et al., 2015; Fiard et al., 2022; Purahong et al., 2019). Cyanobacteria form filamentous biofilms among tree roots (Alvarenga et al., 2015; Wang et al., 2023), which provides a more stable substrate than leaves for biofilm development, and the proximity to the ground provides a high quantity of organic matter, essential for nitrogen-fixing Cyanobacteria (Adame et al., 2014), such as *Pleurocapsa* (15.7% of the root-associated Cyanobacteria here) and *Xenococcus* (14.2%) genera. Interestingly, roots also harbored other less abundant classes such as Planctomycetes, Acidimicrobiia, Anaerolineae, and Chloroflexia, which were entirely absent from the leaf microbiota. Planctomycetia are often reported in mangrove habitats, especially Pirellulales and Planctomycetales in aquatic environments with anoxic sediments (Dedysh et al., 2020). Planctomycetia could contribute to nitrogen fixing and methane and sulfur metabolism (Haldar and Nazareth, 2018), thus promoting plant growth.

### *Avicennia germinans* vs. *Rhizophora mangle*

Strong contrasting patterns were also observed between the ASV profiles of *A. germinans* and *R. mangle*, regardless of the organ analyzed (**Figures 1**, **2**). Both the composition of the root and leaf microbiomes differed significantly between the two species. The root microbiome shared only 3.2% of ASVs between the two tree species, while the leaves shared 1.2%. A significant difference was found between *A. germinans* and *R. mangle* microbial communities for the leaves as well as for the roots (**Table 1**, *p* < 0.05). In particular, while Rhodothermia were reported as a dominant group in *A. germinans*, with up to 30.0% of taxa in the leaves, they represented less than 1.0% of the main taxa for *R. mangle* (**Figure 2**). Notably, the exclusive presence of the class Rhodothermia in the leaves of *A. germinans*, and its absence in *R. mangle*, raises questions about the ecological role of this taxon. Previously reported as major taxa colonizing the *Avicennia* genus (Wainwright et al., 2023), this class could be here specifically recruited on *A. germinans* leaves for their benefit to its growth and health.

**Table 1:** PERMANOVA analysis of bacterial community composition of each compartment, between *A*vicennia *germinans* and *Rhizophora mangle* (999 permutations of the Bray–Curtis matrix).

Compartments	*p*	*r*²	*df*
Leaves	**0.003**	0.065	1
Roots	**0.001**	0.065	1
Sediment	**0.002**	0.065	1
Water	**0.001**	0.065	1

Bold values indicate a significant effect of the tested factors (*p* < 0.05).

Such differences between *A. germinans* and *R. mangle* may have multiple explanations. Physiological and biochemical disparities likely exist between these two species, particularly in the composition of their root exudates (e.g., sugars, organic acids), which serve as substrates or chemical cues for bacteria (D. M. Alongi, 2009; Zhang and Laanbroek, 2020). The leaves also produce species-specific metabolites (antimicrobial or otherwise), such as tannins, phenols, and salts, which may further shape bacterial community composition (Zhang and Laanbroek, 2020). In addition, *A. germinans* eliminates excess salt through its leaves, whereas *R. mangle* does so primarily at the root level. This contrasting strategy may strongly influence their associated microbiomes (Parida and Jha, 2010). There are also marked morphological and anatomical differences between these species: *A. germinans* develops pneumatophores that provide oxygenated niches for certain aerobic microbes, while *R. mangle* produces stilt roots that create more anaerobic microenvironments (Alzubaidy et al., 2016; Holguin et al., 2001). Collectively, these host traits act as ecological filters, leading to species-dependent microbiome specificity, a pattern widely observed across terrestrial and marine ecosystems (Berg et al., 2014).

### Influence of water and sediment on the root microbiome

Given the direct contact between mangrove roots and the surrounding water and sediment, we examined whether this translated into microbiological overlap, specifically whether root-associated microbiomes shared taxa with adjacent aquatic and sedimentary microbial communities. Our results revealed significant differences between the composition of the root microbiome and that of benthic and pelagic communities (**Table 2**, *p* < 0.05), suggesting a likely limited influence of environmental microbes on the structure of the rhizosphere (**Figures 1**, **2**). Roots hosted a dominant proportion of Alphaproteobacteria (39.6%), a group that was comparatively rare in sediment (8%) (**Figure 2**). However, the most important difference was characterized by a high proportion of Gammaproteobacteria in water (21.3%) and sediment (27.9%) and very low in the roots (1.2%). Their high level in the environment appears in line with previous studies emphasizing their contribution to nutrient cycles in mangrove sediments (Haldar and Nazareth, 2018; Meng et al., 2022; Nogueira and Ferreira-Correia, 2001; Yuan et al., 2023). Given their relatively high abundance in both the aquatic environment and root endosphere (Sui et al., 2023; Zhuang et al., 2020), the near absence of Gammaproteobacteria in the rhizosphere of *A. germinans* and *R. mangle* is thus intriguing. Most of the Gammaproteobacteria found in water and sediment belonged to Woeseiaceae (24.9%), whereas this family was largely depleted on tree roots (0.9%). While many Gammaproteobacteria, like Woeseiaceae, are well adapted to aquatic environments such as marine sediments (Mußmann et al., 2017), they could be more affected by the aerial environment of the rhizosphere. The activation of biochemical defense mechanisms, either by the plant itself or by beneficial rhizobacteria, against pathogenic bacteria, such as certain Gammaproteobacteria, could also explain their very low abundance in the root bacteriome of mangrove trees (Kaur et al., 2022).

**Table 2:** PERMANOVA analysis of bacterial community composition between the different compartments of the two mangrove tree species (999 permutations of the Bray–Curtis matrix).

Comparison	*p*	*r*²	*df*
Leaves vs. roots	**0.001**	0.147	1
Leaves vs. sediment	**0.001**	0.154	1
Leaves vs. water	**0.001**	0.165	1
Roots vs. sediment	**0.001**	0.141	1
Roots vs. water	**0.001**	0.151	1

Bold values indicate a significant effect of the tested factors (*p* < 0.05).

Sediment and seawater also harbored a higher number of marginally represented taxa compared to the leaves and roots, such as Acidimicrobiia, Anaerolineae, Desulfobulbia, Verrucomicrobiae, Desulfobacteria, or Campylobacteria (**Figure 2**). In mangrove soils, these groups may play different roles as organic matter degraders or nitrogen and sulfur transformers, such as Acidimicrobiia or Campylobacteria (Silva-Solar et al., 2024; Wang et al., 2024), the latter being also recognized for their pathogenic activity. Their low abundance in the mangrove environment and their absence on trees could be due to a host biotic factor excluding non-beneficial bacteria. This supports the hypothesis of a proper plant-associated microbiome shaped by the trees themselves and not only induced by the availability of bacteria in the environment, as previously demonstrated by Baskaran et al. (2012) and more recently by Elyamine et al. (2023).

## Conclusion

Our findings demonstrate that the mangrove microbiome composition varies markedly between tree organs and across species. They also highlight that while roots are in direct contact with sediment and water, the root microbiome exhibited a distinct taxonomic signature probably shaped by biological, chemical, and ecological filtering, rather than by passive colonization. However, it should also be acknowledged that even if taxonomic overlap was minimal, this does not preclude functional convergence or metabolic exchange between environmental and root-associated communities. Finally, while our study only gives a time snapshot of the microbiome state without taking into account seasonal shifts in the French Guiana mangrove, this work provides elements for further investigations about microbial diversity in the context of changing ecosystems. Further microbiological studies across a broader range of mangrove ecosystems will be needed in the future to improve our understanding of the distribution, diversity, and roles of the mangrove tree-associated bacteria, particularly in the context of climate change.

## Data Availability

The datasets presented in this study can be found in online repositories. The names of the repository/repositories and accession number(s) can be found below: https://www.ebi.ac.uk/ena, PRJEB105373.
